# A bright side facet analysis of borderline personality disorder

**DOI:** 10.1186/2051-6673-1-7

**Published:** 2014-06-11

**Authors:** Adrian F Furnham, John D Crump

**Affiliations:** Research Department of Clinical, Educational and Health Psychology, University College London, London, UK

**Keywords:** Borderline, Traits, Excitable, Neuroticism, Introversion

## Abstract

**Background:**

This study looks at the “bright-side” normal, personality trait correlates of the “dark-side” Borderline Personality Disorder (BPD).

**Methods:**

Over 5000 British adults completed the NEO-PI-R which measures the Big Five Personality factors at the Domain and the Facet level, as well as the Hogan Development Survey which has a measure of Borderline Personality Disorder (BPD) called *Excitable.*

**Results:**

Correlation and regression results confirmed many of the associations between these “bright” and “dark” side individual difference variables. The Excitable score from the HDS was the criterion variable in all analyses. Excitable individuals are high on Neuroticism, but also Introverted and Disagreeable. The facet analysis identified Angry Hostility, Anxiety, Depression and Vulnerability as particularly characteristic of that type.

**Conclusions:**

The study confirmed work on BPD using different population groups and different measures, showing that it is possible to describe personality disorders in terms of extreme scores on personality traits.

## Background

This study looks at “bright side” personality correlates of the “dark side” trait Borderline Personality Disorder (BPD). It examines the relationship between “normal” personality traits measured at the Domain (Super Factor) and Facet (Factor) level, and BPD also called *Excitable.* This study looks at the association between sub-clinical BPD and Domains and Facets of the Big Five personality traits currently the most well used and considered measure and conception of personality in psychological research on personality and individual differences.

For many years there was little rapprochement between psychologists working on personality traits and psychiatrists working on the personality disorders. Some psychologists like Eysenck proposed what is now called *the spectrum hypothesis*, namely that one could understand many mental illnesses in terms of extreme scores on “normal” personality functioning [1]. However there have been significant developments in both fields particularly with differential psychologists and psychiatrists widely accepting the Five Factor Model of personality [2, 3].

### Personality traits and personality disorders

There have been various attempts to integrate ‘normal’ and ‘abnormal’ personality structure and numerous important papers which attempt to link together these systems [3, 4]. Reviews have attempted to set out the possible relationship between the Facets scores from the five factor model and the personality disorders.

An important early review of this relationship was done by Samuel and Widiger [5] who combined the data from 16 studies with a total N of 3207. Most of the participants were students (12 groups) but some were outpatients. Further, they had completed very different personality disorder instruments, yet nearly always the same personality instrument (NEO-PI-R) was used in this study. They analysed the results at both the Domain and Facet level and compared their results to an earlier and similar study by Saulsman and Page [6]. They showed that Borderline was correlated positively with all six Neuroticism Facets (.34 < r < .50). Borderline was also correlated negatively with E1 (Warmth) (r = -.20), E6 (Positive Emotions) (-.26), A1(Trust) (-.29), A2 (Straightforward) (-.21), A4 (Compliance) (-.27), C1 (Competence) (-.29), C3 (Dutifulness) (-.22), C5 (Self Compliance) (-.29) and C6 (Deliberation) (-.27).

Bastiaansen, Rossi, Schotte and De Fruyt [7] compared hypotheses of three reviews. They noted that all three reviewers suggested that BPD people would be high on all Facets of Neuroticism, except N4 (Self Consciousness). There was very little agreement about how the Facets from the other four Domains (Extraversion, Openness, Agreeableness, Conscientiousness) would relate to BPD. Also, they noted that if anything some Facets of Extraversion and Openness (e.g. O3: Feelings) would be positively, and some of Agreeableness (e.g. A1: Trust, A4: Compliance) and of Conscientiousness (e.g. C6: Deliberation) negatively associated with BPD. Bastiaansen et al. [7] also reported data from both a clinical sample of 1029 and a general sample of 659 people. Their results indicated that only two major Domains were involved, namely Neuroticism and Disagreeableness.

Bastiaansen, Rossi and DeFruyt [8] compared five data sets in psychiatric patients. They showed there was complete agreement about the following Facets being linked to BPD: N1, N2, N3, N5, N6, and A4. There is, therefore, little agreement from various sources on the Domain and Facet correlates of BPD other than Neuroticism.

This study attempted to investigate the Domain and Facet correlates of BPD on a large adult sample completing a standard measure of “normal” personality: the NEO-PI-R [9] and the now extensively used Hogan Development Survey [10], which is a measure based on the Personality Disorders categories but useful with normal populations and has an BPD scale [11]. There are at least seven specific BPD measures with varying degrees of psychometric evidence of their validity [12].

### The Hogan development survey

The Hogan ‘dark side’ measure is now extensively used in organisational research and practice to measure personality disorders in the ‘normal population’; [2, 10, 13–15]. Its aim is partly to help selectors and individuals themselves diagnose how they typically react under work stress. The Hogan Development Survey (HDS) was explicitly based on the DSM-IV-TR Axis II Personality Disorder descriptions, but it was not developed for the assessment of all DSM-IV-TR disorders [16, 17]. The HDS focuses only on the core construct of each disorder from a dimensional perspective [18].

An overview of the item selection guidelines can be found in Hogan and Hogan [18]. The HDS has been cross-validated with the MMPI personality disorder scales. Fico, Hogan and Hogan [19] report coefficient alphas between 0.50 and 0.70 with an average of 0.64 and test-retest reliabilities (n = 60) over a three-month interval ranging from 0.50 to 0.80, with an average of 0.68. There were no mean-level differences between sexes, racial/ethnic groups, or younger versus older persons [18]. Various relatively small scale studies have used the HDS and have shown it to be a robust, reliable and valid instrument [13–15, 20].

This study was concerned with the Borderline measure derived from the HDS. The HDS gives scores that are labelled “no risk, low risk, moderate risk and high risk”. The idea is that high scores can be an indicator of business derailment, because under pressure a successful and functioning person may resort to being overly enthusiastic about people or projects, and then becoming disappointed with them. This is seen to have behavioural implications with highly excitable types being energetic and active but moody and irritable; being easily annoyed, and more inclined to give up projects. The excitable types are seen to have difficulty with relationships because of a stop-start, false start approach that leaves people confused about group direction. Very few excitable people are diagnosed with Borderline and can function at high levels in responsible jobs.

The manual [10] provides correlations between the Excitable scale and the 16PF (N = 145). The highest correlations were for Emotional Stability (-.56), Social Boldness (-.37) and Apprehension (.37). It also provides correlations for the IPIP Big 5, 20-item scales (N = 128): Extraversion -.32; Agreeableness, -.20; Conscientiousness -.27; Emotional Stability -.57; and Intellect/Imagination -.14.

This study is concerned with which of the 30 Facets (six for each of the Big Five Personality scores) is related to Excitable. This allows for a better understanding of ‘normal’ BPD or Excitability. Following previous research, it was predicted that (H1) Excitable would be positively correlated with Neuroticism and negatively correlated with Extraversion, Agreeable and Conscientiousness. Next that all Facets of Neuroticism (H2) would be positively and all Facets of Agreeableness (H3) negatively correlated with Excitable.

## Method

### Participants

In total 5726 British working adults took part in this study of which 1213 were females and 4513 males. Their mean age was 42.36 years (*SD* = 7.12 years) with the range being between 23 and 65 years. They were nearly all (over 95%) graduates and in middle class occupations with English as their mother tongue. None, as far as could be established, was diagnosed as BPD.

### Measures

1. NEO-PI-R. The NEO-PI-R [9] is a 240-item inventory, assessing the FFM Domains of Neuroticism (N), Extraversion (E), Openness to experience (O), Agreeableness (A) and Conscientiousness (C), with 6 Facets (8 items each) structured under the Domains. Respondents are requested to provide self-descriptions using a 5-point Likert scale. Its psychometric properties and validity have been well-documented cross-culturally [21]. No item-level information was available for the current sample, but Cronbach Alpha coefficients for the Domains with the Facets as the indicators were .84, .79, .74, .72 and .82 for N, E, O, A, and C respectively.
2. Hogan Development Survey [10] is used in this study. The survey includes 154 items, scored for 11 scales, each grouping 14 items. Respondents are requested to ‘agree’ or ‘disagree’ with the items. The test also has a measure of social desirability. The HDS has been cross-validated with the MMPI personality disorder scales as well as “normal traits” [15]. This study focused on the Excitable scale.

### Procedure

Participants were tested by a British based psychological consultancy over a 10 year period. Each participant was given personal feedback on their score and consented to their anonymous data being published. They were nearly all employed as middle to senior managers in British companies. They took this test as part of an assessment exercise, run by an external psychological consultancy. Inevitably this could have affected their results because of issues such as impression management and dissimulation. However there are two reasons to suspect this did not affect the results. First the HDS has a “lie scale” which can be used to control for this problem. Second, if indeed some dissimilation did occur there is no reason to believe the process would occur differently in males as opposed to females. Ethical approval was sought and gained from the appropriate committee at University College London.

## Results

Correlations and regressions were performed. Excitable correlated with Neuroticism (.37), Extraversion (-.19), Openness (-.02), Agreeableness (-.16) and Conscientiousness (-.19). This supports the first hypothesis (H1). A regression (Table 1) shows that a sixth of the variance could be accounted for primarily through Big Five Neuroticism.

**Table 1:** **Regressions with the** ***Excitable*** **scale as the criterion scale and demographics and the bright side variables as the predictor scales**

	Correlations	Beta	t
Gender	.01	.01	0.18
Age	-.02	-.02	1.14
Neuroticism	.37***	.30	18.36***
Extraversion	-.19***	-.08	4.55***
Openness	-.02	.05	3.21**
Agreeableness	-.16***	-.09	6.58***
Conscientiousness	-.19***	-.01	0.52

F(7, 4933) = 101.68, p < .001, Adj R^2^ = .13.

***p < .001 **p < .01 *p < .0.

Thereafter a regression was performed with the Excitable score as the criterion score and the 30 facet scores as the predictor variables (see Table 2). This also shows correlations with each of the 30 Facets. Most of the Beta weights were significant because of the size of the N. The overall pattern shows that all six Facets of Neuroticism were positively associated with Excitable. This confirms the second hypothesis (H2).

**Table 2:** **Correlations and regression results of excitable (BPD) onto the 30 facets**

Model	Correlations	Beta	t
N1 anxiety	.28	.03	1.97
N2 angry hostility	.35	.19	11.74***
N3 depression	.32	.13	6.95***
N4 self-consciousness	.23	.00	0.09
N5 impulsiveness	.19	.01	0.58
N6 vulnerability	.27	.03	1.83
E1 warmth	-.18	-.06	3.25***
E2 gregariousness	-.19	-.02	1.46
E3 assertiveness	-.16	.01	0.66
E4 activity	-.11	-.02	1.38
E5 excitement-seeking	-.05	-.00	0.06
E6 positive emotions	-.15	-.05	3.26***
O1 fantasy	.05	.02	1.11
O2 aesthetics	-.00	.01	0.87
O3 feelings	.07	.06	4.09***
O4 actions	-.10	-.01	0.65
O5 ideas	-.07	-.03	1.69
O6 values	-.03	.05	3.92***
A1 trust	-.19	-.02	1.49
A2 straightforwardness	-.08	.00	0.42
A3 altruism	-.15	.01	1.19
A4 compliance	-.16	-.02	1.57
A5 modesty	.00	.00	0.64
A6 tender-mindedness	-.07	-.02	1.71
C1 competence	-.18	.03	1.60
C2 order	-.05	.00	0.50
C3 dutifulness	-.15	-.02	1.01
C4 achievement striving	-.14	-.03	1.55
C5 self-discipline	-.20	-01	0.58
C6 deliberation	-.12	-.04	1.80

F(10,6742) = 46.64, Adj R^2^ = .17, p < .001.

***P < .001 **P < .01 * P < .05.

The six facet correlations with Extraversion suggested that Excitable people tended to be Introverted. Four of the six Openness factors were negatively correlated with Excitable, particularly Actions, which is defined as being imaginative, adventurous, optimistic and versatile. Five of the six Agreeableness Facets were negatively related to Excitable, thus confirming H3, but only three had r > .10. All six Conscientious Facets were negatively correlated, particularly Order, Achievement Striving and Deliberation.

Because of potential problems associated with multicollinearity in the analysis shown in Table 2, five further regressions, one for each of the six Facets of the Big Five traits were then done. In each, Excitable was the criterion variable. First, sex, age and were entered, then the six Facets of each of the five factors. The question was which of the six Facets, per Domain.

The regression for the six *Neuroticism* Facets was significant ((F8, 4925) = 92.96, p < .001; Adj R^2^ = .13). Three of the six Facets were significant, the biggest of which were: N2 *Angry Hostility* (Beta .21, t = 13.12, p < .001); N3 *Depression* (Beta .12, t = 5.65, P < .001); and N6 *Vulnerability* (Beta .06, t = 3.09, p < .01).

The regression for the six *Extraversion* Facets was significant ((F(8, 4917) = 24.70; Adj. R^2^ = .04). Three of the six Facets were significant, the biggest of which were E1: *Warmth* (Beta -.12, t = 5.87, p < .001); E2: *Gregariousness* (Beta -.05, t==2.52, p < .01) and E4: *Activity* (Beta .08 , t = 4.58, p < .001).

The regression for the six *Openness* Facets was significant ((F8, 4917) = 16.17, p < .001; Adj. R^2^ = .03). Four of the six Facets were significant, the biggest of which were O1: *Fantasy* (Beta .08, t = 4.79, p < .001); O3: *Feelings* (Beta .07, t = 4.14, p < .001) O4: *Actions* (Beta -.12, t = 7.60, p < .001) and O5: *Ideas* (Beta -.08, t = 4.76, p < .001).

The regression for the six *Agreeableness* Facets was significant ((F8, 4917) = 28.65, p < .001; Adj. R^2^ = .05). Four of the six Facets were significant, the biggest of which were A1: *Trust* (Beta -.12, t = 7.45, p < .001); A3: *Altruism* (Beta -.08, t = 4.86, p < .001); A4: *Compliance* (Beta -.10, t = 5.10, p < .001) and O5 *Modesty* (Beta .05, t = 3.11, p < .01).

The regression for the six *Conscientious* Facets was significant ((F8, 4917) = 27.92, p < .001; Adj. R^2^ = .04). Five of the six Facets were significant, the biggest of which were C1: *Competence* (Beta -.08, t = 4.17, p < .001); C2: *Order* (Beta .08, t = 4.57, p < .001); C4 Achievement Striving (Beta -.04, t = 2.15, p < .05), C5: *Achievement Striving* (Beta -.11, t = 5.18, p < .001) and C6 *Deliberation* (Beta -.05, t = 2.82, p < .01).

## Discussion

The idea of the spectrum hypothesis is that extreme scores on normal personality are an indication of abnormal personality and mental illness. The HDS concept is that most people have a profile of “dark side”, similar to sub-clinical personality traits which at times may well help or more likely hinder them in the workplace [22, 23]. Most people have some “risky dark side traits” but the problem arises when a person comes under pressure or stress and those high-risk (dark) traits become pathological.

This study showed that Excitable people, who may be thought of as sub-clinically BPD, are essentially Neurotic, Disagreeable, Introverts who are low on Conscientiousness. Further, the Facet analysis suggested that Excitable people show Angry Hostility, Depression but little Warmth, Trust, Compliance or Achievement Striving. In this sense they may be thought of as “difficult” colleagues and reports in the work-place.

Most studies using the HDS excitable measure has shown, as predicted, that it is associated with work failure rather than success. Thus Furnham, Trickey and Hyde [24] showed that scores on the Excitable dimension were strongly negatively correlated with measures like managerial Potential (-.54), Reliability (-.42), Stress Tolerance (-.69) and Service Orientation (-.54) (N = 4942). Similarly, Harms, Spain and Hannah [25] studying military cadets over time showed nearly all correlations between peer and superior ratings and the score of Excitable were negative.

The Samuel and Widiger [5] study of students and outpatients (N = 3207) can be compared to the results of this study with “normal adults” (N = 4926) using different BPD measures. The correlations are surprisingly similar given the differences: Neuroticism .54, .37; Extraversion -12, -.19; Openness .10, -.02; Agreeableness -.24, -.16 and Conscientiousness -.29, -.19. In this sense the study provides concurrent evidence for the fact that the Excitable scale is measuring sub-clinical BPD. BPD individuals are thus, what Galen the Greek Philopher, would call Melancholic.

Samuel and Widiger [5] showed all Neuroticism Facets (particularly N3: Depression), two Extraversion Facets (E1: Warmth; E6: positive emotions), no Openness Facets, three Agreeable Facets (N1: Trust; N2: Straightforward, N4: Compliance) and four Conscientiousness factors (C2: Order; C4: Dutifulness, C5: Self Discipline and C6: Deliberation) were correlated r > .20 with BPD. The correlation analyses shown in Table 2 show many similarities, particularly in the highest and lowest correlations of NEO Facets with the Excitable measure. This is interesting given the differences in the samples and the measure of BPD.

Hogan and Hogan [18] devised the HDS to help selection and counselling in the workplace. They noted that Excitable types anticipate being rejected, ignored, criticised, or treated unfairly. They are on guard for signs that others have or will treat them badly and are neither predictable nor rewarding to deal with. As a result they have a lot of trouble building and maintaining a work team. According to the definition and understanding of the Excitable, subclinical BPD can be sensitive to the plight of others; they have some capacity for empathy; because they know that life is not always fair, they can genuinely feel others’ pain. They sometimes tend to be enthusiastic about, and to work very hard on, new projects. They do not handle stress or heavy workloads very well, and they tend to explode rather easily. They are hard people to talk to, and with whom to maintain a relationship. Consequently they change jobs frequently and they have a large number of failed relationships. They are so easily disappointed in working relationships, their first instinct is to withdraw and leave. They are self-centred and all information and experience is evaluated in terms of what it means for them personally and they take the reaction of others personally. They personalise everything, but they do so privately - what others see are the emotional outbursts and the tendency to withdraw. To work with the excitable, managers must be prepared to provide them with a lot of reassurance, keep them well informed so as to minimise surprises, and give them a lot of preview so they know what is coming.

## Conclusions

It is clear from this analysis why Excitable types, at all levels, have difficulty with social relationships and why it is negatively associated with work success. Indeed the personality profile of the Excitable type is almost the exact opposite of what the data suggest is the ideal work profile [2]. Thus the successful leader, worker is Stable, Extraverted, Agreeable and Conscientious while the Excitable person is Unstable, Introverted, Disagreeable and low on Conscientiousness.

This study, like all others, had limitations. There was a problem of multicollinearity with respect to the regressions. It also would be desirable to use a second BPD scale to check the reliability of these findings or indeed have non self-report, like observer data, so as to avoid well known problems associated with reliance on one type of data.
